# Local Mammary Glucose Supply Regulates Availability and Intracellular Metabolic Pathways of Glucose in the Mammary Gland of Lactating Dairy Goats Under Malnutrition of Energy

**DOI:** 10.3389/fphys.2018.01467

**Published:** 2018-10-23

**Authors:** Jie Cai, Feng-Qi Zhao, Jian-Xin Liu, Di-Ming Wang

**Affiliations:** ^1^Institute of Dairy Science, College of Animal Sciences, Zhejiang University, Hangzhou, China; ^2^Ministry of Education Key Laboratory of Molecular Animal Nutrition, Zhejiang University, Hangzhou, China; ^3^Department of Animal and Veterinary Sciences, University of Vermont, Burlington, VT, United States

**Keywords:** glucose supply, metabolic partition, lactation, mammary gland, milk production efficiency

## Abstract

As glucose is the regulator of both the milk yield and mammary oxidative status, glucose supply is considered to play important nutritional and physiological role on mammary gland (MG) metabolism. However, inconsistent results were observed from different infusion methods to evaluate the effect of glucose on MG glucose metabolism. Thus, precise method should be developed to learn how availability and intracellular metabolic pathways of glucose in the MG are altered by the direct mammary glucose supply. In addition, limited information is available on the role of mammary glucose supply in milk synthesis in lactating ruminants under an energy-deficient diet. Direct glucose supply to the MG was implemented in the current study through the external pudendal artery infusion under an energy-deficient diet. Six doses of glucose (0, 20, 40, 60, 80, and 100 g/d) were infused through the external pudendal arteries, which is the main artery to the MG, to six lactating goats fed with basal diet meeting 81% energy requirement in a 6 × 6 Latin square design. Milk and lactose yields were both quadratically increased with increased glucose infusion, whereas the milk yield changed inconsistently with the increased energy balance (EB), indicating local glucose supply, rather than EB, improved milk production. Glucose fluxes in the MG were significantly increased and correlated with mammary plasma flow. However, the ratio of lactose yield to glucose absorbed by the MG was significantly decreased. The increased glucose fluxes in the MG and changed glucose-related metabolites in milk indicated that the glucose availability and intracellular metabolic pathways was regulated by local mammary glucose. Acute glycolysis consumed the superfluous glucose and induced accumulation of oxygen radicals in the MG during over-supplied glucose conditions. The present study provided insight to optimal glucose supply to the MG during the lactation.

## Introduction

Milk production is largely influenced by precursor supply to the mammary gland (MG) (Toerien et al., 2010). Glucose is one of the most important nutrients for milk production as it is the major substrate of lactose synthesis (Zhao, 2014). Enhanced lactose synthesis is directly linked to milk yield because of its osmotic regulation of milk volume (Liu et al., 2013). The nicotinamide adenine dinucleotide phosphate (NADPH) derived from glucose is also required for the synthesis of fatty acids and proteins (Bauman and Griinari, 2003). Thus, the MG of high-yielding lactating ruminants is in great demand of glucose to maintain active milk synthesis.

Numerous studies have investigated the effect of glucose supply on lactation performance (Rulquin et al., 2004; Lemosquet et al., 2009; Wang et al., 2016b). A positive effect has been demonstrated on milk yield with increased doses of glucose infusion through abomasum (Huhtanen et al., 2002). In some studies, however, glucose did not stimulate milk yield when infused ruminally or intravenously (Vanhatalo et al., 2003; Curtis et al., 2014). The inconsistency may be at least partially attributed to the sites when glucose is infused and thus, the glucose availability to the MG in these studies (Curtis et al., 2014; Nichols et al., 2016). In fact, more than 40% of metabolizable glucose in the blood may not be captured by the MG for milk synthesis (Sunehag et al., 2003). Mammary glucose availability is influenced by the post-intestinal-absorptive bioprocesses (Hurtaud et al., 2000), such as glucose utilizations by the skeletal muscle, liver or other catabolic pathways (Sciascia et al., 2010). In addition, glucose is also partitioned within cells, which includes the partition of glucose between lactose synthesis, glycolysis, the pentose phosphate pathway (PPP), or other metabolic pathways in mammary epithelial cells (MECs) (Zhao, 2014; Zachut et al., 2016). Glucose availability to the MG and their metabolic partition within MECs play an important role in regulating glucose utilization efficiency in the MG.

In addition, previous studies mainly focused on the effect of infused glucose on milk production and MG metabolism in lactating animals under energy sufficient conditions (Rulquin et al., 2004; Lemosquet et al., 2009; Nichols et al., 2016). However, in developing countries, lactating animals are often fed in the energy-deficient conditions, such as feeding rice straw or corn stover as forage sources. Thus, there is also a need to study the effect of glucose supply to the MG on milk production in energy-deficient conditions.

Precise nutrition, which takes accurate nutrition management into consideration at the levels of metabolism, cells, and organs, works based on the knowledge of the dose-dependent effects of individual nutrients and local nutritional requirements in the specific organ. The optimal glucose supply, referring to minimal dose of glucose to achieve maximal milk production, has not been ascertained yet for the dairy ruminants. Studies *in vitro* provided clues with the optimal concentrations of glucose to promote the synthesis of lactose and other milk component at cellular aspect (Liu et al., 2013; Lin et al., 2016). However, little information is available on the optimal glucose supply to milk production and the mechanisms that control intracellular mammary glucose partition to milk synthesis *in vivo*. Recent studies have applied infusion techniques to accurately offer different glucose supplies (Hurtaud et al., 2000; Huhtanen et al., 2002). However, the infusion sites were mainly carried out at the rumen, abomasum, or duodenum, which compromise the accurate assessment of the true glucose availability in the MG due to the involvement of the bioprocesses of digestion, glucose absorption and post-absorptive partition.

Local mammary infusion can be an accurate way to investigate the optimal glucose supply for the MG and an effective way to evaluate how glucose metabolism responds to the glucose availability in MG (Cant et al., 2002; Xiao and Cant, 2003). By using this technology, in the present study we investigated the effects of local mammary glucose supply on milk performance and mammary glucose utilization in lactating dairy goats fed a diet which met 81% of energy requirement. The local glucose supply was manipulated by glucose infusion into the external pudendal artery (EPA), which is the main artery to the MG. The optimal glucose supply was determined by fitting curves of the milk production, lactose synthesis, and mammary glucose fluxes observed in infused animals.

## Materials and Methods

### Animals and Experimental Design

All animal experimental procedures used in this study were approved by the Animal Use and Care Committee of Zhejiang University (Hangzhou, China). Six second-lactation dairy goats [body weight: 43.6 ± 3.0 kg (mean ± SD), days in milk: 113 ± 6, and milk yield: 1.47 ± 0.05 kg/d] were used in this study. Goats were fitted with catheters at the EPAs as described below and assigned to one of six doses of glucose infusion in a 6 × 6 Latin square design with repeated measures of over 12d periods, including 7d treatment and 5d transition. The goats were milked at 3 time points (0630, 1000, and 1900 h) with a bucket milker. All goats were fed the same basic ration containing forages and pelleted concentrates at 0700, 1100, and 1730. The experimental diet (Table 1) was formulated based on the NRC recommendations for dairy goats to meet 81% of energy requirement (National Research Council, 2007). Six doses of glucose [0 (G0), 20 (G20), 40 (G40), 60 (G60), 80 (G80), and 100 (G100) g/d] were infused through the installed catheter for 7 d during each treatment period.

**Table 1:** Ingredient and chemical compositions of the basal diet (dry matter basis).

Item	Amount	*SD*
**Ingredient, g/kg**		
Alfalfa hay	200.0	–
Peanut cane	160.0	–
Chinese wildrye	320.0	–
Corn grain	154.0	–
Wheat bran	64.0	–
Soybean meal	38.0	–
Canola meal	26.0	–
Salt	16.0	–
CaHPO_4_	6.0	–
Premix^∗^	16.0	–
**Composition, % of dry matter**		
Dry matter	88.4	2.1
Organic matter	90.9	3.3
Crude protein	11.3	1.1
Ether extract	3.1	0.2
Acid detergent fiber	24.1	2.5
Neutral detergent fiber	34.3	3.1
Non-fiber carbohydrate	42.8	1.4
NE_L_, MJ/kg^†^	4.7	0.4


*^∗^Premix composition: 65 g Mg/kg, 3.2 g Cu/kg, 6.5 g Fe/kg, 27.8 g Mn/kg, 25.6 g Zn/kg, 35 mg Se/kg, 78 mg Co/kg, 0.3 g I/kg, 55 g S/kg, 2000 kIU vitamin A/kg, 350 kIU vitamin D_*3*_/kg, 2.5 g vitamin E/kg, 10 g nicotinic acid/kg, and 20 g choline/kg. ^†^Calculated data. NE_*L*_, net energy for lactation.*

### Catheterization and Infusions

Catheterization was performed 4 weeks before the infusion. Before the catheterization procedures, animals were fasted and deprived of water for 24 and 12 h, respectively. Goats were anesthetized with xylazine hydrochloride (0.50 mg/kg). After the surgical cuts in the groin areas, silicone catheters (1.20 mm in outer diameter and 0.60 mm in inner diameter, Shanghai Fengcheng Rubber Products Co., Shanghai, China) were inserted into the EPA of both sides (extended 15 cm into the EPA). The free end of the catheters passed through the skin and was fixed to the back of the rumps. The catheters were filled with heparinized saline solution (200 IU heparin sodium/ml saline) and capped with a heparin cap during maintenance.

Glucose was weighted and fully dissolved in 600 mL of saline solution. The pH of each glucose solution was adjusted to 7.30 to 7.40 and then filtered through a 0.45-μm filter. During each treatment day, glucose solutions were infused continuously from 1200 to 1700 at a speed of 2.00 mL/min into the EPAs with syringe infusion pumps (Smiths WZS-50F6, Smiths Medical Instrument, Zhejiang, China).

### Measurement, Sampling, and Analyses

During the treatment time of each period, feed and orts were weighed daily. Feed offered was adjusted to allow for 5% orts. Dry matter intake was calculated based on the feed and orts offered and consumed. Feed samples were collected daily for the analysis of nutrient compositions as listed in Table 1.

In each period, milk yield was recorded for successive 3 d. Meanwhile, 50 mL of milk was taken daily at 3 time points (0630, 1000, and 1900 h) and pooled for determination of milk composition (fat, protein, lactose, total milk solids, milk urea nitrogen, and somatic cell count), glucose-related metabolites [glucose, glucose-6-phosphate (G6P), citrate, and lactic acid] and oxidative stress-related indexes [malondialdehyde (MDA), reactive oxygen species (ROS) and oxygen radical antioxidant capacity (ORAC)]. Milk compositions were analyzed with an automatic ultrasonic milk composition analyzer (Bentley Instruments, Minnesota, United States). Glucose-related metabolites and oxidative stress-related indexes were determined using the commercial kits (Meibiao Biotechnology Co., Jiangsu, China). Another 10 mL of milk was collected at 3 time points (0630, 1000, and 1900 h) and stored at -20°C for later analysis of phenylalanine (Phe) and tyrosine (Tyr) in milk.

The EPA blood samples (5 mL) were collected from the both sides of catheters at -5, 0, 0.25, 0.5, 0.75, 1, 1.5, 2, 2.5, 3, 4, and 13 h relative to the finishing time of daily infusion in the last day of each period. The blood samples taken from each side at each individual time point were pooled, and the pooled blood samples were immediately centrifuged at 3000 × g for 15 min at 4°C and stored at -80°C until analysis for blood physiological and biochemical parameters using an Auto Analyzer 7020 (Hitachi High-Technologies Corporation, Tokyo, Japan) and hormones. Another set of blood samples (5 mL) was taken at 3 time points (0630, 1000, and 1900 h) from the both sides of EPAs and mammary veins separately, and pooled according to sample sites after centrifugation for further analysis of physiological and biochemical parameters in the plasma, and amino acids (Phe and Tyr).

The pooled plasma were treated by the method described previously (Mackle et al., 1999) and analyzed for amino acids (AAs) using an Automatic AA Analyzer (Hitachi High-technologies Corporation, Tokyo, Japan). Milk was pretreated with the acid hydrolysis method (Delgadoelorduy et al., 2002) and then analyzed for AAs using the Automatic AA Analyzer. The blood concentrations of insulin and glucagon in the EPA and mammary vein were analyzed using the commercial kits from the Meibiao Biotechnology Co., Jiangsu, China.

### Calculations and Statistical Analysis

The energy balance (EB) was calculated as follows (Hammon et al., 2009):

EB (MJ/d) = NE_L_ intake – (ECM × 3.14 + 0.293 × kg of BW^0.75^) + infused glucose,

where NE_L_ represents net energy for lactation, ECM represents energy-corrected milk yield (kg/d) and was calculated by 0.3246 × milk yield + 13.86 × milk fat yield + 7.04 × milk protein yield, and infused glucose provided 11.51 Mcal/kg of NE_L_. The mammary plasma flow (MPF) was calculated by Fick’s principle (Mepham, 1982; Cant et al., 1993). The equation was as follows:

MPF (L/d) = (milk Phe + Tyr) (g/d) × 0.965/[Arterio-venous difference of (Phe + Tyr) (g/L)]

Indices reflecting glucose fluxes in MG were calculated as follows:

Mammary arterial supply of glucose (g/d) = Gluc_A_ (g/L) × MPF (L/d)

Mammary venous outflow of glucose (g/d) = Gluc_V_ (g/L) × MPF (L/d)

Arterio-venous difference (AVD, g/L) = Gluc_A_ (g/L) - Gluc_V_ (g/L)

where, Gluc_A_ and Gluc_V_ indicate the arterial and venous glucose concentration, respectively.

Indices reflecting glucose utilization by MG were calculated as follows:

Mammary uptake of glucose (g/d) = AVD (g/L) × MPF (L/d)

Clearance rate of glucose (L/h) = AVD (g/L) × MPF (L/h)/Gluc_V_ (g/L)

To assess insulin resistance, the surrogate index was calculated according to Balogh et al. (2008) by the equation:

RQUICKI_BHB_ = 1/[log glucose (mg/dL) + log insulin (μU/mL) + log NEFA (mmol/L) + log BHB (mmol/L)]

where RQUICKI_BHB_ represents the revised quantitative insulin sensitivity check index, BHB represents β-hydroxybutyric acid, and NEFA represents non-esterified fatty acid. A lower value suggests a greater insulin resistance.

All data were evaluated for normal distribution before statistics using the Data Processing System software (Tang and Zhang, 2013). All data were analyzed using the MIXED model (SAS 9.21) with a 6 × 6 Latin square design. Treatment, goat, period, and residual effects were considered as the sources of the variation. The treatment and period were considered as fixed variables. The goat was considered to be the random variable. The residual effect was used to test the significance of treatment, goat, and period. Differences for treatments were analyzed by orthogonal polynomial contrasts of linear and quadratic effects. Results were present as least square means with mean square errors. Interaction between infused glucose, milk production, and EB was analyzed using the correlation analysis (SAS 9.21). Time-caused dynamic analysis of glucose concentration and its related hormones were additionally taken time effect and treatment effect into account. Regression analysis was implemented by fitting curve (Origin 8) and the slope of the curve was calculated by its first-order derivative. The *P* values of linear and quadratic effects were reported as *P_linear_* and *P*_quadratic_, respectively. For dynamic analysis, *P*-values of time and treatment effects were reported as *P_time_* and *P*_treatment_, respectively. Significance was defined as *P* < 0.05 and tendency was defined as 0.05 < *P* < 0.10.

## Results

### Feed Intake, EB and Milk Performance

**FIGURE 1:**
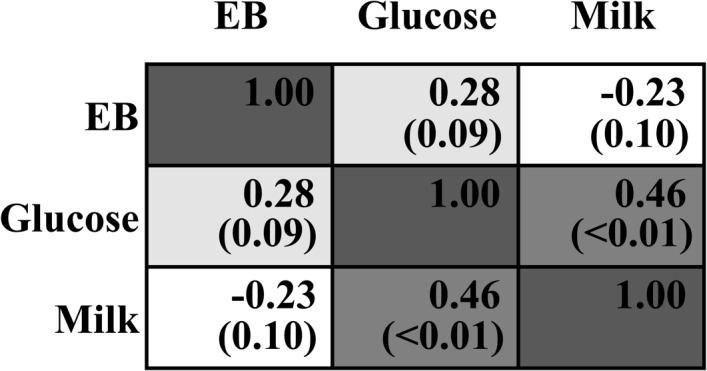
Correlation analysis between infused glucose level (Glucose), energy balance (EB), and milk production (Milk). The upper number within each cell means Pearson Correlation Coefficient. The lower number with the bracket means *P*-value.

Dry matter intake was not different across all treatments (*P* > 0.05, Table 2). EB was significantly changed from negative when 0–60 g/d of glucose was infused to positive when 80–100 g/d was infused (*P_linear_* < 0.01, *P*_quadratic_ < 0.01), with no difference among G0 to G60 groups and between G80 and G100 groups. Milk yield and ECM increased in a quadratic manner in response to mammary glucose supply (*P*_quadratic_ < 0.01). Correlation analysis showed no interaction between infused glucose and EB (R = 0.28, *P* = 0.09, Figure 1) or between milk production and EB (R = -0.23, *P* = 0.10), whereas a significant interaction between infused glucose and milk production (R = 0.46, *P* < 0.01). The yield of lactose and fat and lactose content also showed a quadratically increased response (*P*_quadratic_ < 0.01). Milk urea nitrogen content was linearly decreased with increasing mammary glucose supply (*P_linear_* < 0.01). The yield and content of lactose and milk yield were the highest when glucose was infused at the level of 60 g/d.

**Table 2:** Effects of increasing mammary glucose supply by external pudendal artery on feed intake, energy balance, and milk production.

Item^∗^	Infused glucose (g)						SEM	*P*-value^†^	
	0	20	40	60	80	100		L	Q
Dry matter intake, kg	1.65	1.67	1.66	1.62	1.67	1.60	0.05	0.31	0.45
Energy balance, MJ/d	–0.18^b^	–0.14^b^	–0.30^b^	–0.43^b^	0.50^a^	0.47^a^	0.17	<0.01	<0.01
Milk yield, g	692^c^	750^bc^	813^a^	880^a^	776^b^	758^bc^	26.1	0.04	<0.01
ECM, g	938^c^	1,018^bc^	1,115^ab^	1,166^a^	1,025^bc^	1,037^bc^	37.5	0.09	<0.01
Fat yield, g	37.8^b^	41.5^ab^	45.9^a^	46.7^a^	41.5^ab^	41.5^ab^	2.07	0.27	<0.01
Protein yield, g	27.0	28.4	30.4	33.0	28.0	30.7	1.51	0.12	0.13
Lactose yield, g	33.3^c^	35.6^bc^	38.5^ab^	41.0^a^	36.4^bc^	35.1^bc^	1.26	0.20	<0.01
**Milk composition, %**					
Fat	5.46	5.52	5.68	5.33	5.36	5.45	0.26	0.65	0.87
Protein	3.88	3.79	3.76	3.75	3.61	4.07	0.17	0.78	0.14
Lactose	4.64^c^	4.70^bc^	4.75^ab^	4.81^a^	4.74^ab^	4.65^c^	0.03	0.24	<0.01
TSC	14.7	14.9	14.9	14.7	14.5	14.9	0.29	0.82	0.85
MUN, mgN/dL	33.9^ab^	36.3^a^	33.2^b^	32.0^bc^	30.0^c^	29.2^c^	0.71	<0.01	0.08
SCC ×10^3^/mL	797^ab^	647^ab^	209^b^	463^b^	1,009^a^	1,018^a^	242	0.20	0.03


*^∗^ECM, energy-corrected milk yield; TSC, total solid content; MUN, milk urea nitrogen; SCC, somatic cell count. ^†^L, linear effect; Q, quadratic effect. Values (such as a,b,c) in the upper-right corner differ (*P* < 0.05) if without a common letter.*

### Dynamic Changes of Blood Variables

**FIGURE 2:**
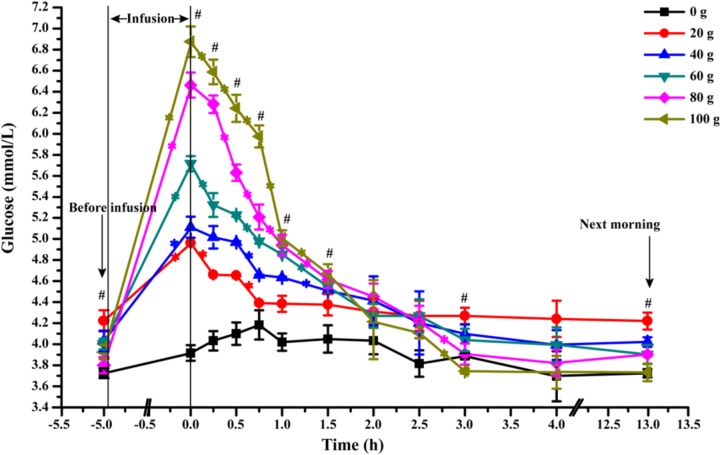
Dynamic changes of artery glucose concentration after infusion of different doses of glucose (0–100 g/d) through external pudendal artery. The 0 time in the X axis represents the end time of infusion. “^∗^” represents significant difference in the time effect in each treatment, and “^#^” represents significant difference in the treatment effect at individual time points (*P* < 0.05).

The dynamic changes of artery glucose concentration before and after glucose infusion was presented in Figure 2. At the end of infusion, artery glucose concentration increased linearly from 0 to 100 g/d of glucose infusion (*P_linear_* < 0.01). The glucose concentration decreased rapidly within 1.5 h after infusion in all glucose infusion treatments (average slop of dynamic glucose concentration curves within 1.5 h was -0.99) and at slower rates between 1.5 and 3 h (average slop was -0.35) after infusion (*P_time_* < 0.01). All the glucose infusion groups reached a steady EPA glucose concentration after 3 h (average slop was -0.05, *P_time_* < 0.01). However, the declining slopes of artery glucose after infusion were different among different glucose dose treatments. Goats in the G100 (average slop was -1.81) and G80 (average slop was -1.51) groups had the steepest negative slopes among the treatment groups within 60 min (*P_time_* < 0.01, *P*_treatment_< 0.01), showing a rapid glucose partition among the circulatory system. During 3 to 13 h, the area under curve of G80 (38.60) and G100 (37.33) groups were lower when compared with that of G20 (42.33), G40 (40.10), and G60 (39.53) groups (*P* < 0.01).

The concentrations of blood variables at 13 h after glucose infusion, when the artery glucose concentration became stable in each treatment, were presented in Table 3. In the artery, glucose concentration was quadratically increased with increasing glucose supply (*P*_quadratic_ < 0.01). In contrast, BHB concentration was linearly decreased (*P_linear_* < 0.01). No differences were shown in NEFA (*P* > 0.05) and triglyceride (*P* > 0.05) concentrations in the artery across all the treatments. In the vein, these blood variables remained relatively stable with increased glucose supply, except that there was a tendency of quadratic increase in glucose (*P*_quadratic_ = 0.09). The AVD of glucose was not significantly different across all groups (*P* > 0.05).

**Table 3:** Effects of increasing mammary glucose supply by external pudendal artery on physiological and biochemical parameters in artery and vein at 13 h after glucose infusion (steady period).

Item^∗^	Infused glucose (g)						SEM	*P*-value^†^	
	0	20	40	60	80	100		L	Q
**Artery**									
Glucose, mmol/L	3.72^b^	4.22^a^	4.02^a^	3.90^ab^	3.90^ab^	3.73^b^	0.046	0.52	<0.01
BHB, umol/L	421^a^	418^a^	422^a^	385^b^	385^b^	372^b^	10.8	<0.01	0.47
NEFA, umol/L	150	159	173	204	162	153	19.4	0.73	0.09
Triglyceride, mmol/L	0.157	0.164	0.110	0.145	0.135	0.128	0.029	0.42	0.70
**Vein**									
Glucose, mmol/L	3.11	3.57	3.34	3.28	3.11	3.25	0.085	0.30	0.09
BHB, umol/L	147	151	173	159	134	141	11.2	0.31	0.10
NEFA, umol/L	271	238	211	325	195	264	44.9	0.89	0.81
Triglyceride, mmol/L	0.131	0.132	0.101	0.121	0.098	0.105	0.030	0.40	0.83
**AVD**									
Glucose, mmol/L	0.610	0.647	0.682	0.722	0.693	0.673	0.039	0.13	0.14
BHB, mmol/L	273^a^	267^ab^	249^abc^	226^c^	251^abc^	230^bc^	13.5	0.02	0.43


*^∗^BHB, β-hydroxybutyric acid; NEFA, non-esterified fatty acid; AVD, arterio-venous difference. ^†^L, linear effect; Q, quadratic effect. Values (such as a,b,c) in the upper-right corner differ (*P* < 0.05) if without a common letter.*

### Mammary Blood Flow and Glucose Flux

**FIGURE 3:**
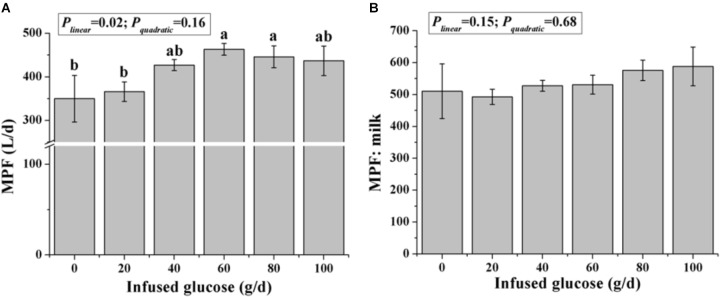
Effects of increasing mammary glucose supply (0–100 g/d) through the external pudendal artery on mammary plasma flow [MPF, **(A)**] and the ratio of mammary plasma flow and milk volume **(B)**. Error bar represents mean of standard error.

The MPF was significantly increased with increasing glucose infusion from 0 to 60 g/d and was not increased with glucose infusion over 60 g/d (*P_linear_* < 0.05, *P_quadratic_* > 0.05, Figure 3A). The MPF value per unit of milk was not different among all treatments (*P* > 0.05, Figure 3B).

Mammary glucose uptake was linearly increased with increasing mammary glucose supply (*P_linear_* = 0.02, Table 4). The glucose clearance rate was significantly increased (*P_linear_* = 0.02, Table 4), but the glucose extraction rate was not significantly changed (*P_linear_* = 0.12, Table 4) with increasing glucose supply. However, the mammary uptake, and clearance rate of glucose were not increased when more than 60 g/d of glucose was infused. At 60 g/d of infusion, the mammary uptake, and clearance rate of glucose were 52.2% and 43.8% higher, respectively, than those in the control animals receiving no glucose infusion.

**Table 4:** Effect of increasing mammary glucose supply through external pudendal artery on glucose utilization in the mammary gland.

Item	Infused glucose (g)						SEM	*P*-value^†^	
	0	20	40	60	80	100		L	Q
Mammary arterial supply of glucose, g/h	9.77^b^	11.79^ab^	12.86^ab^	13.93^a^	12.73^ab^	12.92^a^	1.05	0.03	0.07
Mammary venous out flow of glucose, g/h	8.12^b^	10.03^ab^	10.68^a^	11.43^a^	10.39^ab^	10.71^a^	0.871	0.05	0.07
Mammary uptake of glucose, g/d	39.5^b^	42.0^b^	52.3^ab^	60.1^a^	56.2^ab^	53.3^ab^	5.36	0.02	0.10
Clearance rate of glucose, L/h	2.97^b^	2.67^b^	3.63^ab^	4.27^a^	4.21^a^	3.78^ab^	0.417	0.02	0.22
Extraction rates, %	0.164	0.154	0.169	0.181	0.182	0.172	0.010	0.12	0.51


*^†^L, linear effect; Q, quadratic effect. Values (such as a,b,c) in the upper-right corner differ (*P* < 0.05) if without a common letter.*

**FIGURE 4:**
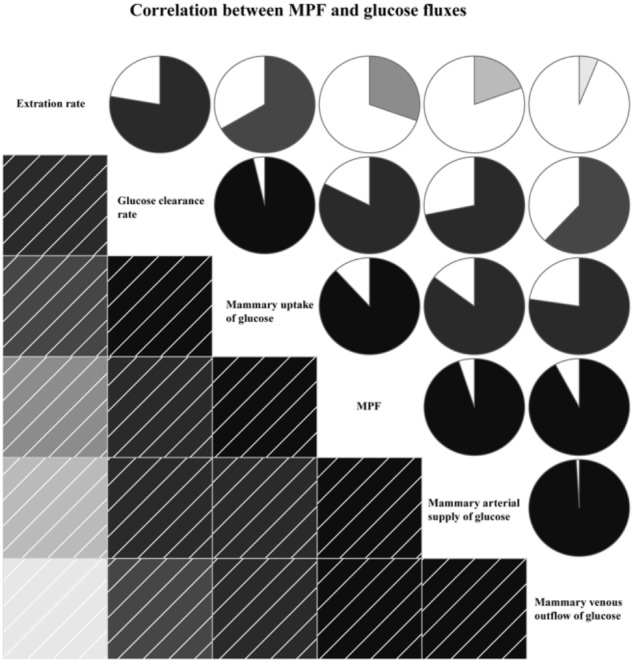
Correlation between mammary plasma flow (MPF) and glucose fluxes. In left square matrix, the deeper color and the higher the saturation of the cells, the greater positive correlation of variables. Meanwhile, in right circle matrix, the filled pie charts from clockwise visualize the positive correlation of variables.

The MPF was positively correlated with glucose fluxes, including mammary arterial glucose supply (R = 0.95, *P* < 0.01), mammary venous outflow of glucose (R = 0.92, *P* < 0.01), glucose uptake (R = 0.88, *P* < 0.01), glucose clearance rate (R = 0.82, *P* < 0.01), and extraction rate (R = 0.30, *P* < 0.01, Figure 4).

### Glucose Utilization by Mg

Increasing glucose supply from 0 to 60 g/d increased mammary arterial supply of glucose (*P_linear_* = 0.03, Table 4) and mammary venous outflow of glucose (*P_linear_* = 0.05), but mammary arterial supply of glucose and mammary venous outflow of glucose was not increased when > 60 g/d glucose was infused.

**FIGURE 5:**
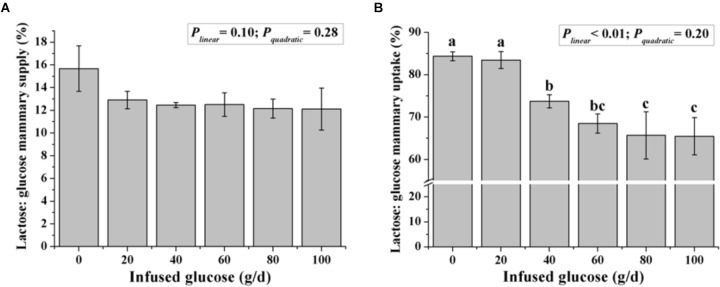
Effect of increasing mammary glucose supply through the external pudendal artery on synthesis rate of lactose [the ratio of lactose yield and glucose mammary supply **(A)** and the ratio of lactose yield and glucose mammary uptake **(B)**]. Error bar represents mean of standard error.

Although the ratio of lactose to mammary glucose supply was not changed with increasing glucose supply (*P_linear_* = 0.10, Figure 5), the ratio of lactose to mammary glucose uptake, an indicator of glucose metabolic partition intracellularly, declined from 84.4 to 65.5% when glucose was infused from 0 to 100 g/d (*P_linear_* < 0.01).

**FIGURE 6:**
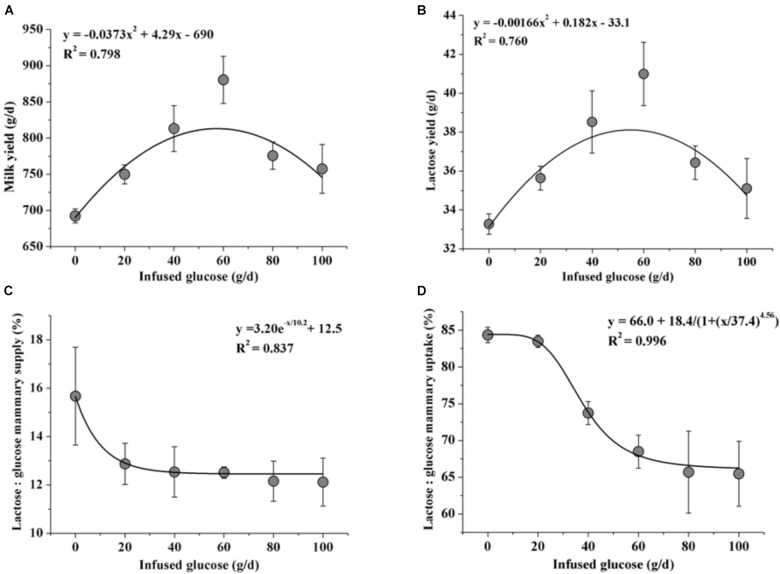
Fitting curves of milk production [milk yield **(A)** and lactose yield **(B)**] and glucose utilization [the ratio of lactose yield and glucose mammary supply **(C)** and the ratio of lactose yield and glucose mammary uptake **(D)**] when 0–100 g/d of glucose was infused to the mammary gland. Error bar represents standard error.

Fitting curve analysis was used to estimate the optimal glucose supply in goats (Figure 6). The analyses of milk yield and lactose yield in response to glucose infusion showed that the 50 to 60 g/d of glucose infusion to the MG maximized the milk production. However, there were drastic declines in ratios of lactose to mammary glucose supply and lactose to mammary glucose uptake when over 40 g/d of glucose was infused.

Milk indexes related to glucose metabolism and oxidative stress in MG were presented in Table 5. With increasing glucose supply, concentrations of milk glucose (*P_quadratic_* = 0.03) and ORAC (*P_quadratic_* = 0.05) was quadratically increased, whereas milk G6P (*P_quadratic_*< 0.01) and ROS (*P_quadratic_*< 0.01) was quadratically decreased. The milk glucose and ORAC both reached the maximum value at the glucose infusion of 60 g/d, whereas the milk G6P and ROS both reached the maximum value at the glucose infusion of 100 g/d. The ratio of G6P and glucose in milk tended to decrease quadratically with increasing glucose supply (*P_quadratic_* = 0.07). Milk lactic acid was linearly increased (*P_linear_* = 0.02). Milk MDA tended to decrease (*P_linear_* = 0.07). Milk citrate was not significantly different between different glucose supplies.

**Table 5:** Effect of increasing mammary glucose supply through external pudendal artery on glucose metabolites in milk.

Item^∗^	Infused glucose (g)						SEM	*P-*value^†^	
	0	20	40	60	80	100		L	Q
Glucose, μmol/L	51.27^b^	52.27^b^	55.49^ab^	59.92^a^	54.01^ab^	53.70^b^	3.66	0.22	0.03
G6P, μmol/L	52.93^ab^	52.03^ab^	51.20^bc^	49.26^c^	52.93^ab^	53.78^a^	0.74	0.43	<0.01
G6P/glucose	1.05	1.00	0.95	0.83	1.01	1.05	0.09	0.87	0.07
Citrate, mmol/L	6.60	6.77	6.99	6.92	7.01	6.87	0.64	0.67	0.69
Lactic acid, μmol/L	3.62^b^	3.63^ab^	3.82^ab^	3.83^ab^	3.89^ab^	3.94^a^	0.11	0.02	0.78
Malondialdehyde, nmol/L	15.57	15.41	14.41	14.66	14.32	14.63	0.52	0.07	0.28
ROS, mg of H_2_O_2_/100 mL	13.21^ab^	12.80^bc^	11.60^cd^	10.73^d^	13.81^ab^	14.32^a^	0.47	0.06	<0.01
ORAC, U/mL	9.80^ab^	10.02^ab^	10.04^ab^	10.56^a^	9.53^ab^	9.12^b^	0.38	0.19	0.05


*^∗^G6P, glucose-6-phosphate; ORAC, oxygen radical antioxidant capacity. ^†^L, linear effect; Q, quadratic effect. Values (such as a,b,c) in the upper-right corner differ (*P* < 0.05) if without a common letter.*

### Dynamic Changes of Blood Insulin and Glucagon

**FIGURE 7:**
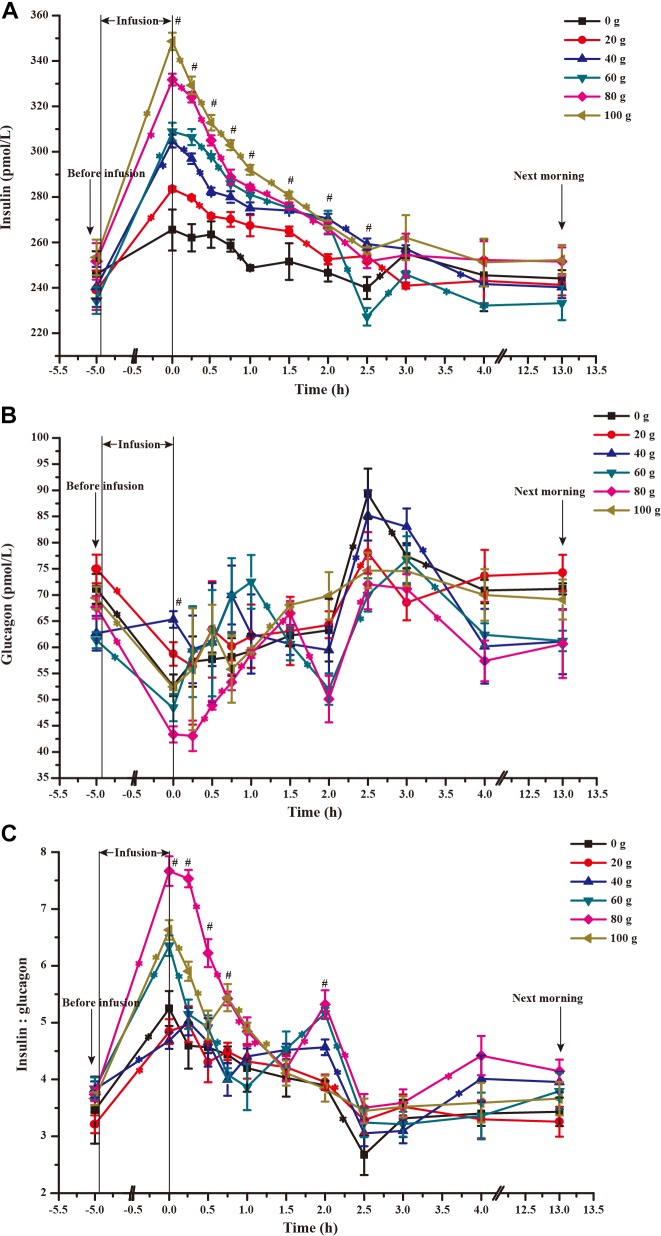
Time-course of artery insulin **(A)** and glucagon **(B)** and their ratio **(C)** when 0–100 g/d of glucose was infused to the mammary gland. The 0 time in the X axis represents the end time of infusion. “^∗^” represents significant difference in time effect of each group, and “^#^” represents significant difference in treatment effect at individual time points (*P* < 0.05).

**FIGURE 8:**
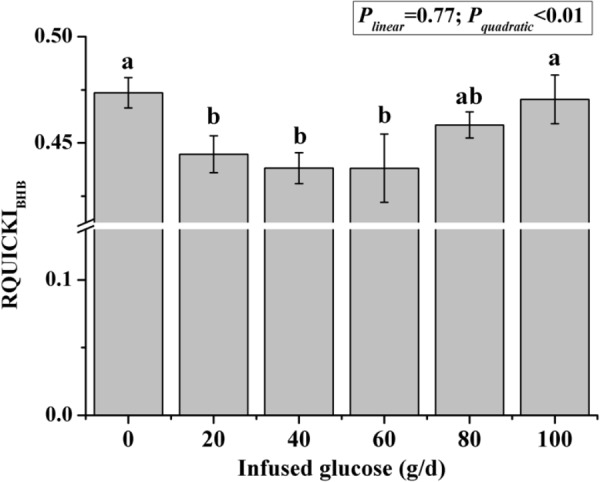
Revised quantitative insulin sensitivity check index corrected by β-hydroxybutyric acid (RQUICKI_BHB_) in goats with increasing mammary glucose supply through the external pudendal artery. Error bar represents standard error.

The time courses of artery insulin and glucagon are shown in Figure 7. Glucose infusion induced insulin surge in blood in all groups. For all groups, glucose infusion-induced insulin secretion declined in the first 3 to 4 h after the infusion (Figure 7A). However, goats receiving 80 and 100 g/d of glucose had higher blood insulin concentrations at the end of infusion compared to other groups (*P* < 0.01, treatment effect). Insulin response parameters were calculated to evaluate the different insulin responses in each treatment (Table 6). With increased glucose supply, the peak (at 0 h, *P*_linear_ = 0.01) and increment of insulin (*P*_linear_ = 0.02) were linearly increased in accordance with the significantly increased area under the curve in 240 min after infusion (*P*_linear_ = 0.05), indicating a sustained high level of blood insulin in high glucose supply (G80 and G100) compared with low glucose supply (G0 and G20). The RQUICKI_BHB_ was significantly decreased in a quadratic manner (*P*_quadratic_ < 0.01, Figure 8) and was the lowest at 40 or 60 g/d of glucose infusion. Thus, the concentration and sensitivity of insulin could be the vital regulator of glucose fluxes and MG utilization.

**Table 6:** Time-course response indexes of blood insulin and glucagon to different doses of glucose infusion.

Item^∗^	Infused glucose (g)						SEM	*P*-value^†^	
	0	20	40	60	80	100		L	Q
**Insulin, pmmol/L**									
Ct0	246	239	240	235	252	253	7.3	0.25	0.13
Peak	278^b^	283^b^	305^ab^	309^ab^	332^a^	348^a^	21.5	0.01	0.76
Increment	32.3^b^	43.9^b^	64.8^ab^	74.1^ab^	79.8^ab^	94.3^a^	20.8	0.02	0.80
t basal, min	112	134	106	136	142	126	7.6	0.06	0.47
AUC240	6030^b^	6175^b^	6433^ab^	6317^ab^	6557^ab^	6687^a^	247	0.05	0.95
**Glucagon, pmmol/L**									
Ct0	71.3	74.9	62.8	61.6	67.7	69.4	6.48	0.52	0.25
Nadir	52.9	58.7	59.4	48.6	43.4	52.5	4.52	0.10	0.85
Decrement	–18.4	–16.2	–3.4	–12.9	–24.3	–16.8	8.76	0.79	0.27
t basal, min	112^a^	113^a^	113^a^	55^c^	92^b^	115^a^	9.50	0.19	0.01
AUC240	1638	1598	1644	1587	1441	1618	63.0	0.25	0.59


*^∗^Ct0, basal concentration before infusion; Peak, peak concentration; Increment, Peak – Ct0; Nadir, nadir concentration; t basal, time to reach basal concentration; AUC240, area under the curve in the 240 min after infusion, pmmol/dL^∗^240min. ^†^L, linear effect; Q, quadratic effect. Values (such as a,b,c) in the upper-right corner differ (*P* < 0.05) if without a common letter.*

Dynamic change of blood glucagon showed an inverse relationship with insulin (Figure 7B), but the glucagon response parameters, including basal concentration before infusion, nadir, decrement, and area under the curve in 240 min after infusion were not significantly different among treatments (*P* > 0.05). However, the time to reach basal concentration showed significant quadratic response (*P*_quadratic_ < 0.01).

Ratio of blood insulin to glucagon was higher in the G80 and G100 groups within 45 min after infusion (*P*_treatment_ < 0.01), but it declined significantly within 90 min (*P_time_* < 0.01) and did not differ from other treatments in the other time periods (Figure 7C).

## Discussion

### Local Mammary Glucose Supply, EB and Milk Synthesis

Lactation is a biological function to extend life for next generation. Adequate amounts of glucose are essential for MG to maintain high milk yield as both the energy source and the main precursor of lactose (Zhao, 2014). Even through, there are knowledge gaps in this filed, such as what the optimal glucose supply is and how the glucose will exhibit the metabolic priority between energy supply and lactose synthesis under the energy-deficient condition. Previous studies have reported that glucose had a positive effect on lactation emphasizing the role of glucose metabolism under sufficient energy condition (Huhtanen et al., 2002; Rius et al., 2010). However, under the energy-deficient condition in this study, it seemed that lactation still had a metabolic priority on the glucose metabolism over the changing of EB because of a quadratic increased milk yield and lactose yield with no difference in EB between the infusion levels of 0 g/d to 60 g/d glucose. During the onset of lactation, metabolic priority of milk production is observed from different adaptive reactions of supplying energy and substrate for the MG at the expense of the lactation-induced NEB and illness (Gross, 2012). Interestingly, even when lactating animals were under the energy-deficient conditions, strategies of nutrient partitioning were still likely to provide substrates for milk production, such as increasing mammary glucose uptake in a quadratic manner. Some studies showed negative or no effects of glucose supply on lactation under energy-sufficient conditions (Vanhatalo et al., 2003; Curtis et al., 2014). However, the negative effects of glucose were also observed at the high glucose infusion (G80 and G100) under energy-deficient condition. Inconsistency of the previous studies and the current one in the effect of glucose on lactation and the quadratic response of milk yield, lactose yield and mammary glucose uptake in the current study indicated that glucose had a dose-dependent effect on the lactation and prefer to satisfy the metabolic allocation for lactation rather than to change the EB condition.

Moreover, the EB changes caused by different levels of glucose infusion do not appear to be a cause for changes in lactation performance. When glucose infusion was lower than 60 g/d, EB was negative; whereas when the infusion level was over 60 g/d, EB became positive. However, the milk and lactose yields were actually decreased when EB changed from negative in G60 group to positive in G80 and G100 groups. Furthermore, EB showed a decreasing tendency when glucose was infused from 0 to 60 g/d, but the milk yield was increased in these animals. Thus, these discrepancies between milk performance and EB indicated that the milk production changes associated with the infused glucose levels was resulted from the substrate supply, but not from EB differences, even when animals were in negative EB. Moreover, significant correlation was found between the infused glucose level and milk production, but not between the infused glucose level and EB or between milk production and EB. This may be explained by that unlike in non-ruminant animals, glucose is not the major energy source in ruminant animals (Bergman, 1990; Barcroft et al., 1994), however, glucose is the major precursor of lactose synthesis which controls milk volume by maintenance of milk osmolality (Zhao, 2014).

However, whereas the milk production increased when glucose was infused to 60 g/d, the glucose utilization efficiency linearly declined with increasing glucose supply. A level of 50 to 60 g/d extra glucose supply to MG is considered to be optimal for both high lactation performance and relatively high glucose utilization efficiency in goats with the current experimental conditions. The specific reasons for the depressed efficiency in the higher dose infusion treatments are discussed below.

### Mammary Glucose Availability of Glucose in Response to its Supply

Glucose precursor generation, gluconeogenesis in liver, and glucose transport mechanisms in the MEC were intensively studied (Zhao and Keating, 2007; Li et al., 2013; Zhang et al., 2015). However, few studies have focused on how mammary glucose availability is affected by local glucose infusion. Only glucose taken up by MG can be used for milk synthesis, therefore the glucose availability to MG is particularly important.

The function of infused glucose and its partition can be deduced from the phases of blood glucose changes after infusion, including the rapid dynamic phase (Figure 2: 0–90 min after glucose infusion), slow descent stage (Figure 2: 90–180 min after glucose infusion), and the steady-state phase (Figure 2: 180 min after glucose infusion to next infusion). A fast glucose partition seemed to occur within 90 min after infusion, when glucose is quickly distributed and absorbed among tissues. Liver and skeletal muscle glycogen synthesis likely plays a major role in this phase. Tissue glucose absorption appears to become slower during slow descent phase in 90 to 180 min after infusion. During the steady phase, blood glucose becomes stable as the glucose production from gluconeogenesis and glycogen hydrolysis reached a balance with glucose utilization by tissues.

Interestingly, according to the area under curves, the artery glucose concentration in the G80 and G100 groups became even lower than that of G20, G40, and G60 after 180 min of infusion (Figure 2), which probably led to lower amounts of glucose consumed by the MG. The higher venous outflow of animals in G80 and G100 compared to the animals in G0 showed that large amounts of glucose were released into the venous circulatory system as unutilized by the MG. Glucose fluxes (arterial supply of glucose, mammary uptake of glucose, clearance rate of glucose) were elevated at the optimum glucose infusion (G60) for high mammary glucose utilization, in which a higher glucose was maintained in the MG circulation. Thus, these data indicated that glucose supply to the MG within a proper range enhances glucose fluxes in the MG and glucose availability to MG. The MG has a predominant role in the glucose availability from circulation when glucose was supplied at a moderate level (Rigout et al., 2002). Excess supply of glucose to the MG reduces the glucose available to the MG.

The main factors regulating glucose availability in the MG are MPF and hormones (Sano et al., 1991; Madsen et al., 2015). The metabolites in the MG, which rely on the nutrients available to MG from the blood, can affect MPF (Cant et al., 2016). A previous study suggested that insufficient availability of glucose decreased MPF and mammary glucose uptake by 30% (Wang et al., 2016a). Consistently, the optimum glucose supply increased MPF by 32%, and excess glucose supply decreased MPF by 6% in our current study. These results suggested that a nutrient-driven change in blood flow rate is a potential mechanism to regulate glucose availability to the MG. A strong correlation between MPF and glucose flux indexes, including arterial glucose supply, venous outflow of glucose, glucose uptake, and glucose clearance rate, was observed in this study (Figure 4), further supporting the critical roles of MPF in mammary glucose availability by influencing the glucose fluxes in the MG.

Endocrine hormones play critical roles in regulating body metabolism. Insulin is the most important regulator in glucose metabolism (Nemazanyy et al., 2015). It is known that insulin indirectly regulates mammary glucose supply through regulation of glucose uptake in the peripheral tissues, mainly skeletal muscle and adipose tissue (Singh et al., 2014). In this study, the changes in blood insulin and insulin response parameters (peak and area under the curve in 240 min after infusion) showed that insulin levels remained high for 240 min after infusion, which likely resulting in high glucose absorption by peripheral tissues. In addition, the calculated insulin sensitivity index (RQUICKI_BHB_) implied that the increasing glucose supply curvilinearly increased insulin resistance in goats. It appeared that the adipose cells might reduce the over-absorbing extra glucose to convert into lipids, reflected by low levels of BHB, in certain levels of glucose supply. Thus, optimal glucose supply may maintain a high ratio of glucose allocated to MG by inducing insulin resistance in peripheral tissues. However, insulin resistance may be overtaken by high levels of insulin resulting from excess glucose supply in G80 and G100.

Glucagon is another important hormone for glucose metabolism in the circulatory system (Jiang and Zhang, 2003). In this study, the glucagon response parameters, such as the time to reach basal concentration, showed that the appropriate glucose supply requires high levels of glucagon. Glucagon may stimulate gluconeogenesis to enhance glucose availability to the MG.

### Metabolic Partition of Glucose in the MG

After glucose is absorbed by the MG, most of them (60–85%) are used to synthesize lactose (Nielsen and Jakobsen, 1993). However, lactose synthesis is greatly dependent on the glucose partition ratio in the metabolic pathways within the MECs, including lactose synthesis, glycolysis, PPP, and the tricarboxylic acid cycle (Zhao, 2014).

Our observations on mammary arterial supply of glucose, mammary venous outflow of glucose, and the ratio of lactose to glucose mammary uptake in this study indicated that an increasing glucose supply might alter the mammary glucose partition between lactose synthesis and other pathways. Along with the increasing glucose supply, the utilization of glucose in lactose synthesis decreased from 84.4 to 65.5%, implying that glucose might be shifted from lactose synthesis to other pathways. Milk glucose, G6P, lactic acid, MDA, ROS, and ORAC were then analyzed to provide information on the metabolic fate of the intracellular glucose. These analyses showed that: (i) the ratio of G6P and glucose was lowest in the optimal glucose infusion groups (G40 and G60), and a G6P/glucose ratio > 1 in milk, which was caused by recycling of fructose-6-phosphate formed in the PPP to G6P (Stincone et al., 2015), was observed in both the treatment with low-supplied (G0 and G20) and over-supplied (G80 and G100) glucose, indicating that a proportion of glucose was shunted to PPP and formation of NADPH; (ii) lactic acid increased with increasing glucose supply, indicating increased glycolysis; and (iii) higher ROS levels in the low-supplied and over-supplied glucose treatments, as well as lower ORAC levels in relative with optimal glucose treatments implied both low-supplied and over-supplied glucose may cause oxidative stress in MG. The causatives of higher ROS and lower ORAC under low-supplied and over-supplied glucose conditions were probably different. In the animals with low dose of glucose (G0 and G20), lipolysis and a large supply of artery BHB compensated for the negative EB in G0 and G20 (Table 3), thus lower ORAC likely resulted from the lipid peroxidase derived from the high BHB. This notion was supported by high MDA of milk in these groups. On the other hand, when glucose was over-supplied, the lower ORAC could be resulted from the high production of ROS derived from superfluous glucose (Eleftheriadis et al., 2017). From these information, it is suggested that the MG demonstrated a degree of metabolic flexibility toward different glucose supply and modified the balance between the lactation and oxidative stress.

### Conclusion, Perspectives, and Significance

Our study employed a local mammary glucose intervention by EPA infusion to investigate how MG responds to increased local glucose availability in energy deficient condition. Our results showed that the local glucose supply has a dose-dependent effect on milk performance and mammary glucose utilization by changing mammary glucose availability and metabolic pathways of glucose within MECs. We also found that acute glycolysis consumes the superfluous glucose, and oxygen radicals accumulate in MG during excessive glucose conditions. Furthermore, MPF, insulin, and glucagon may play important roles in glucose partition and utilization in the MG. The study provided new insights into glucose utilization efficiency in lactation and dairy nutrition.

## Author Contributions

JC, J-XL, and D-MW designed the research. JC and D-MW conducted the experiments and analyzed the data. JC, D-MW, F-QZ, and J-XL wrote the paper; and D-MW had primary responsibility for the final content. All authors read and approved the final manuscript.

## Conflict of Interest Statement

The authors declare that the research was conducted in the absence of any commercial or financial relationships that could be construed as a potential conflict of interest.

## Abbreviations

AA: amino acids
AVD: arterio-venous difference
BHB: β-hydroxybutyric acid
EPA: external pudendal artery
G6P: glucose-6-phosphate
MDA: malondialdehyde
MG: mammary gland
MPF: mammary plasma flow
NADPH: nicotinamide adenine dinucleotide phosphate
NEFA: non-esterified fatty acid
ORAC: oxygen radical antioxidant capacity
Phe: phenylalanine
PPP: pentose phosphate pathway
RQUICKI: revised quantitative insulin sensitivity check index
Tyr: tyrosine
